# Altered resting-state brain functional activities and networks in Crohn’s disease: a systematic review

**DOI:** 10.3389/fnins.2024.1319359

**Published:** 2024-01-24

**Authors:** Ling Yang, Peipei He, Lingqin Zhang, Kang Li

**Affiliations:** ^1^Radiology Department, Chongqing General Hospital, Chongqing, China; ^2^Department of Radiology Children’s Hospital of Chongqing Medical University, National Clinical Research Center for Child Health and Disorders, Ministry of Education Key Laboratory of Child Development and Disorders, Chongqing, China

**Keywords:** Crohn’s disease, fMRI, brain activity, functional connectivity, systematic review

## Abstract

**Background:**

Crohn’s disease (CD) is a non-specific chronic inflammatory disease of the gastrointestinal tract and is a phenotype of inflammatory bowel disease (IBD). The current study sought to compile the resting-state functional differences in the brain between CD patients and healthy controls.

**Methods:**

The online databases PubMed, Web of Science Core, and EMBASE were used to find the published neuroimage studies. The search period was from the beginning through December 15, 2023. The predetermined inclusion and exclusion criteria allowed for the identification of the studies. The studies were assembled by two impartial reviewers, who also assessed their quality and bias.

**Results:**

This review comprised 16 resting-state fMRI studies in total. The included studies generally had modest levels of bias. According to the research, emotional processing and pain processing were largely linked to increased or decreased brain activity in patients with CD. The DMN, CEN, and limbic systems may have abnormalities in patients with CD, according to research on brain networks. Several brain regions showed functional changes in the active CD group compared to the inactive CD group and the healthy control group, respectively. The abnormalities in brain areas were linked to changes in mood fluctuations (anxiety, melancholy) in patients with CD.

**Conclusion:**

Functional neuroimaging helps provide a better understanding of the underlying neuropathological processes in patients with CD. In this review, we summarize as follows: First, these findings indicate alterations in brain function in patients with CD, specifically affecting brain regions associated with pain, emotion, cognition, and visceral sensation; second, disease activity may have an impact on brain functions in patients with CD; and third, psychological factors may be associated with altered brain functions in patients with CD.

## Introduction

The inflammatory bowel diseases (IBD) are chronic inflammatory disorders affecting the gastrointestinal tract, including Crohn’s disease (CD) and ulcerative colitis (UC) (Sands, 2015; Gracie et al., 2019). Histopathologically, CD is characterized by irregular, localized, and full-thickness inflammation throughout the whole gastrointestinal system, while UC is characterized by widespread inflammation limited to the mucosal layer of the colon. As a more serious type of disease, the symptoms of CD are manifested as abdominal pain, diarrhea, bloody stool, weight loss, long-term complications (abscesses, fistulae, and strictures), and extraintestinal manifestations (affected joints, skin, eyes, and other organs), which seriously affect the quality of life of patients (Bakshi et al., 2021; Rogler et al., 2021). Approximately 25% of patients with CD develop symptoms before age 20 (Baldassano and Piccoli, 1999). A recent systematic review pointed out that the prevalence was highly variable and was rising rapidly in newly industrialized countries, such as Asia and Latin America (Kaplan and Windsor, 2021). It was estimated that in the next 30 years, the incidence rate of CD and ulcerative colitis in these countries should approximate the coalescing incidence range in the western world: 12–26 per 100,000 people (Kaplan and Windsor, 2021).

Although the pathophysiology of CD is not fully understood, several factors might contribute to CD, including genetic susceptibility, environmental factors, intestinal dysbiosis, and dysregulated mucosal immune responses (Sartor, 2006). In recent years, the relationship between psychological morbidity and inflammatory activity has aroused interest. The incidence of CD in combination with psychological comorbidity, including stress, anxiety, and depression, has been estimated at up to 35% (Neuendorf et al., 2016). The brain–gut axis is frequently used to explain the complex mechanisms between neuroendocrine pathways, the peripheral, central, and autonomic nervous systems, and the gastrointestinal tract (Bonaz and Bernstein, 2013). It describes how symptoms of CD affect brain structure and function, and how these changes can impact the neural substrate of the complex interactions between psychological comorbidity, pain, and gastrointestinal functions (Aziz and Thompson, 1998; Bernstein, 2017). Conversely, brain dysfunction could affect the digestive system by mediating the release of adrenocorticotropic hormone and increasing intestinal permeability through the axis. It is important to further understand the alterations in brain function related to CD to elucidate the pathophysiology.

Nowadays, the development of blood oxygenation level-dependent functional magnetic resonance imaging (BOLD-fMRI) has allowed non-invasive detection of brain activity changes. The categories of inspection techniques are task state and resting state (rs-fMRI). Rs-fMRI is typically used to detect changes in BOLD associated with minimal physical or mental activity in the absence of task design (Raimondo et al., 2021). Analytical methods are comprised of amplitude of low-frequency fluctuation (ALFF) (Zou et al., 2008), regional homogeneity (ReHo) (Zang et al., 2004), functional connectivity (FC) (Roy et al., 2009), etc. Using rs-fMRI, several studies have reported brain dysfunction involved in the default mode network (DMN) (Thomann et al., 2017; Hou et al., 2019), central executive network (CEN) (Hou et al., 2019; Li L. et al., 2021), and limbic regions (Fan et al., 2019) in patients with CD. These brain networks are considered to modulate pain perception, affect, and environmental factors on gut function, and their function abnormality may lead to visceral hypersensitivity and intestinal inflammatory responses. Task-state fMRI is typically used to detect changes in brain activity based on specific tasks or events (Glover, 2011). By performing a Stroop color-word interference task, Agostini et al. (2013, 2017) reported that the stressful task in CD patients elicited abnormal brain activities in the amygdala, hippocampus, insula, putamen, cerebellar regions, and midcingulate cortex (MCC). They pointed out that the dysfunction of these brain regions in patients with CD may dysregulate the autonomic stress-evoked responses and neuroendocrine systems and abnormally integrate emotional processes with sensory information input from the gut (Agostini et al., 2013, 2017). However, due to the inconsistency of research methods, multi-network interactions, and inconsistent research conclusions, the interaction between changes in brain dysfunction and gut characteristics in patients with CD is still largely unclear.

In the current study, we conducted a systematic review to identify the abnormalities in resting-state brain functions and their association with psychological comorbidity and abdominal pain in patients with CD, respectively.

## Materials and methods

This study was conducted according to the preferred reporting items for systematic reviews and meta-analyses (PRISMA) 2020 statement (Page et al., 2021) and followed the recommendations for neuroimaging meta-analysis (Nichols et al., 2017). No published protocol existed for this study.

### Search strategy

The Ovid, PubMed, and Web of Science databases were searched systematically by 2 researchers, respectively, from their inceptions to December 15th, 2023, using the keywords: “Crohn’s disease,” “Magnetic Resonance Imaging,” “functional MRI,” “resting-state fMRI,” etc. Supplementary material showed the specific search strategies. Ethical approval was not necessary, as this study was a systematic review based on published studies.

### Eligibility criteria

Inclusion criteria for studies were as follows: (1) articles were written in English; (2) patients were diagnosed with CD; (3) patients with CD and healthy controls (HCs) were above 18 years old; and (4) imaging technology for brain screening was not limited. Exclusion criteria for studies were as follows: (1) studies were non-population (e.g., cell lines, animal studies); (2) literature types were abstract, letter, review, or other non-research article, (3) studies were brain structure only; (4) duplicate literature; (5) sample size was small (CD cases less than 10); (6) studies were lack of HCs; and (7) task-state studies.

### Data selection and collection

Ling Yang searched for literature in the appropriate data library according to the search formula. After removing duplicates, Ling Yang and Peipei He screened the titles and abstracts to remove literature unrelated to the topic of the systematic review. The remaining studies were reviewed in full text and identified based on the inclusion criteria. The disagreement between two researchers was solved by discussion. Ling Yang extracted the basic characteristics of the included study, including lead author, year, country, imaging technique, analysis methods, sample size, subject characteristics, and main results. The references to the retrieved articles were manually searched for additional studies. Missing data were tried to be obtained from the authors.

### Risk of bias assessment

Lingqin Zhang and Peipei He evaluated the quality and risk of bias of the included literature based on Nichols et al.’s (2017) study. There were six items that needed to be evaluated: research objectives, recruitment, eligible criteria, population demographics, imaging methodology, and comparison group. If the included studies met all six items, they would be evaluated as low risk; if they met five items, they would be evaluated as medium risk; and if they met four items or less, they would be evaluated as high risk.

## Results

**Figure 1:**
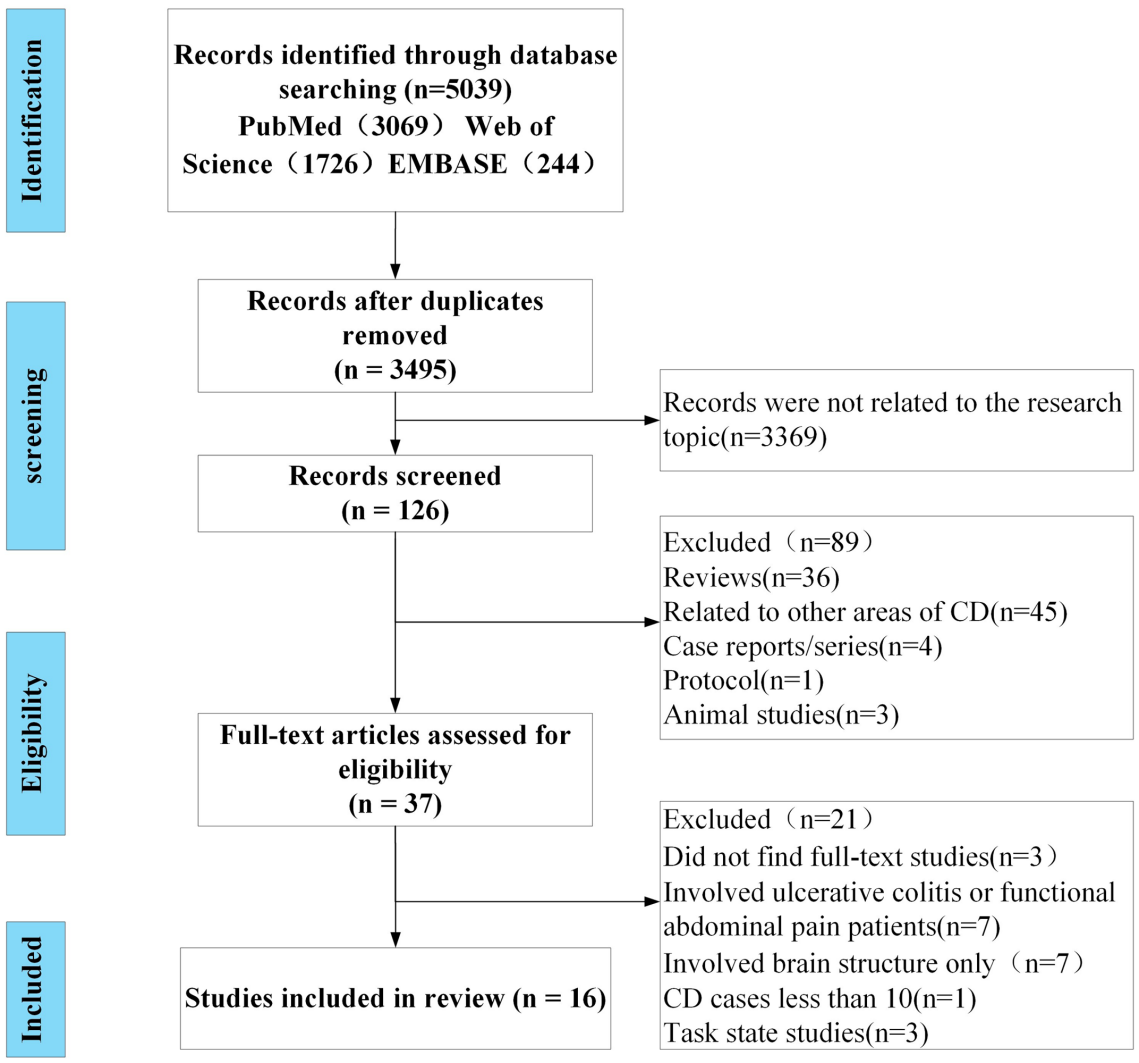
The flow diagram of the review.

A total of 5,039 studies were retrieved from three databases, after removing duplicate literature, 3,495 studies remained. Most articles (*n* = 3,369) were excluded after reading the title, as they were not related to the main issues discussed in this system review. Of the remaining 126 studies, we removed 89 inappropriate studies (36 reviews, 45 studies related to other areas of CD, 4 case reports or series, 1 protocol, and 3 animal studies). Of the 37 potential studies, the full text of 3 studies were not available, 7 studies involved ulcerative colitis or functional abdominal pain patients, 7 studies involved brain structure only, 3 studies involved task state, and another study was excluded because of CD cases less than 10. Finally, a total of 16 eligible original studies were included in this study, and the flowchart of literature screening and qualification review was shown in Figure 1.

### Study characteristics

We analyzed 16 original studies published in neuroscience, gastroenterology, and multidisciplinary journals from 2013 to 2023, including a total of 548 patients with CD and 502 healthy controls. Eleven studies come from China, one from the United States, two from Italy, one from Germany, and one from Canada. For CD categories, 9 studies included inactive CD patients, 1 study included active CD patients, 4 studies included active CD patients and inactive CD patients, and 2 studies did not mention the categories of patients. One study explored CD with or without pain versus HCs. Table 1 summarized the study’s characteristics.

**Table 1:** Study characteristics of included Crohn’s disease studies.

Author, year	Country	Imaging technique	Analyze methods	Sample size (CD/HCs)	Sex in CD (Male/Female)	Comparison	Matched parameters between groups
Bao C. et al. (2016)	China	rsfMRI	ReHo	25/25/36	16/9 19/6	CD with pain vs. CD without pain vs. HCs	Age, sex, BMI
Bao C. H. et al. (2016)	China	rsfMRI	ReHo	52/36	37/15	CD vs. HCs	Age, sex, BMI
Thomann et al. (2017)	Germany	rsfMRI	FNC	15/14	6/9	CD vs. HCs	Age, sex, education, BMI, cardiovascular factors
Bao et al. (2018)	China	rsfMRI	ALFF; ROI-wise FC	60/40	43/17	CD vs. HCs	Age, sex, BMI, education
Liu et al. (2018)	China	rsfMRI	Topological analysis	43/37	28/15	CD vs. HCs	Age, sex, BMI, education
Hou et al. (2019)	United States	rsfMRI	FNC	18/18	10/8	CD vs. HCs	Age, sex, education
Fan et al. (2019)	China	rsfMRI	ROI-wise FC	42/35	27/15	CD vs. HCs	Age, sex, BMI
Kornelsen et al. (2020)	Canada	rsfMRI	FNC	35/21	18/17	CD vs. HCs	Age, sex, BMI
Li J. et al. (2021)	China	rsfMRI	ALFF; ReHo; ROI-wise FC	15/26	11/4	CD vs. HCs	Age, sex, education
Kong et al. (2022)	China	rsfMRI	ALFF	15/19/20	9/6 13/6	active CD vs. inactive CD vs. HCs	Age, sex
Li et al. (2022)	China	rsfMRI	FNC	20/22	8/12	CD vs. HCs	Age, sex, handedness, and education
Qiu et al. (2022)	China	rsfMRI	ROI-wise FC	22/22	12/10	CD vs. HCs	Age, sex, education
Huang et al. (2022)	China	rsfMRI	ReHo	58/57/92	47/11 44/13	active CD vs. inactive CD vs. HCs	Age, sex, handedness, and education
Zhang et al. (2022)	China	rsfMRI	ROI-wise FC	45/40	25/20	CD vs. HCs	Age, sex
Agostini et al. (2023)	Italy	rsfMRI	FNC	19/14/18	7/12 6/8	active CD vs. inactive CD vs. HCs	Age, sex, education
Thapaliya et al. (2023)	Italy	rsfMRI	FNC	25/25	16/9	active CD vs. HCs	Age, sex, BMI

fMRI, functional magnetic resonance; rs-fMRI, resting-state functional magnetic resonance; BOLD, blood oxygenation level-dependent; ReHo, regional homogeneity; FC, functional connectivity; FNC, functional network connectivity; ALFF, amplitude of low frequency fluctuation; ROI, Region of interest; CD, Crohn’s disease; HCs, health controls; BMI, body mass index.

### Study quality and risk of bias

Lingqin Zhang and Peipei He evaluated the quality and risk of bias of the included literature based on the six items and reached an agreement. Among all studies, 15 were evaluated as having a low risk of bias. One study was evaluated as having a medium risk of bias because of the lack of detailed recruitment and eligibility criteria. Details can be seen from the Table 2.

**Table 2:** The quality and risk of bias for studies.

Author, year	Research objectives	Recruitment	Eligible criteria	Population demographics	Imaging methodology	Comparison group	Risk of bias
Bao C. et al. (2016)	Y	Y	Y	Y	Y	CD with pain vs. CD without pain vs. HCs	Low
Bao C. H. et al. (2016)	Y	Y	Y	Y	Y	CD vs. HCs	Low
Thomann et al. (2017)	Y	N	Y	Y	Y	CD vs. HCs	Medium
Bao et al. (2018)	Y	Y	Y	Y	Y	CD vs. HCs	Low
Liu et al. (2018)	Y	Y	Y	Y	Y	CD vs. HCs	Low
Hou et al. (2019)	Y	Y	Y	Y	Y	CD vs. HCs	Low
Fan et al. (2019)	Y	Y	Y	Y	Y	CD vs. HCs	Low
Kornelsen et al. (2020)	Y	Y	Y	Y	Y	CD vs. HCs	Low
Li L. et al. (2021)	Y	Y	Y	Y	Y	CD vs. HCs	Low
Kong et al. (2022)	Y	Y	Y	Y	Y	Active CD vs. inactive CD vs. HCs	Low
Li et al. (2022)	Y	Y	Y	Y	Y	CD vs. HCs	Low
Qiu et al. (2022)	Y	Y	Y	Y	Y	CD vs. HCs	Low
Huang et al. (2022)	Y	Y	Y	Y	Y	Active CD vs. inactive CD vs. HCs	Low
Zhang et al. (2022)	Y	Y	Y	Y	Y	CD vs. HCs	Low
Agostini et al. (2023)	Y	Y	Y	Y	Y	Active CD vs. inactive CD vs. HCs	Low
Thapaliya et al. (2023)	Y	Y	Y	Y	Y	Active CD vs. HCs	Low

CD, Crohn’s disease; HCs, health controls.

### Brain functional activities at rest

Brain functional activities at rest may reflect the blood oxygenation level-dependent (BOLD) signals obtained from the time series collected when subjects are in slow breathing and minimal physical or mental activity (Rosazza and Minati, 2011). The analytical methods include the ReHo and ALFF. It is believed that the brain’s functional activity and spontaneous fluctuations are reflected in the ALFF (Zhang and Li, 2010). Additionally, another alternative indicator of the brain spontaneous function is ReHo. We summarized six studies that employed ALFF or ReHo. Compared to HCs, patients with CD showed hypoactivity at resting-state in:(1) frontal lobe, including the medial prefrontal cortex (mPFC), supplementary motor area (SMA), inferior frontal operculum cortex, and precentral gyrus (Bao C. et al., 2016; Bao C. H. et al., 2016; Bao et al., 2018; Huang et al., 2022); (2) parietal lobe, including precentral gyrus and S2 (Bao C. et al., 2016; Bao et al., 2018); (3) temporal lobe; including superior temporal cortex and middle temporal cortex (Bao C. et al., 2016; Huang et al., 2022); (4) insula (Bao C. et al., 2016); (5) limbic lobe and nucleus, including the median cingulate cortex (MCC), posterior cingulate cortex (PCC), amygdala, thalamus and hippocampal/parahippocampal cortex, periaqueductal gray (PAG) (Bao C. et al., 2016; Li L. et al., 2021). Hyperactivity was found in: (1) the frontal lobe, including the superior frontal cortex, middle frontal cortex, and SMA (Bao C. et al., 2016; Bao C. H. et al., 2016; Bao et al., 2018; Li L. et al., 2021; Huang et al., 2022; Kong et al., 2022); (2) the parietal lobe, including the precuneus, angular gyrus, and superior parietal cortex (Bao C. et al., 2016; Bao et al., 2018); (3) the temporal lobe, including the inferior temporal cortex and middle temporal cortex (Bao C. et al., 2016); (4) insula (Bao C. H. et al., 2016; Bao et al., 2018); (5) limbic lobe and nucleus, including the ACC, MCC, and hippocampal/parahippocampal cortex (Bao C. et al., 2016; Bao C. H. et al., 2016; Bao et al., 2018; Li L. et al., 2021; Kong et al., 2022). Clinical characteristics and psychological evaluation were assessed using the Crohn’s disease activity index (CDAI), Hospital Anxiety and Depression Scale (HADS), Visual Analog Scale (VAS), Social Support Rating Scale (SSRS), etc. In these studies, HADS-D scores were positively correlated with mWavelet-ALFF values of the left ACC (Kong et al., 2022). The psychological assessments of objective support scores were positively correlated with the ReHo values of the right frontal superior medial brain regions in active CD patients and negatively correlated with the right postcentral and supplementary motor area brain regions (Huang et al., 2022). The ReHo values of the left frontal middle brain regions were positively correlated with the depression, obsessive-compulsive, and bigoted scores and negatively correlated with the systemic symptoms score in these CD patients (Huang et al., 2022).

### Brain functional connectivities at rest

Resting-state functional connectivity (rs-FC) refers to correlations in the low-frequency spontaneous fluctuations of BOLD signals across brain regions (Biswal et al., 1995). Region of interest (ROI) -wise FC analysis makes it possible to identify networks spanning the entire brain, identify regional changes within a cortical area, and determine individual variances between subjects (Braga and Buckner, 2017). In this review, five studies were conducted on region of interest (ROI) -wise FC analysis. Regarding ROI selection, we concluded that the ROI was primarily located in the bilateral hippocampus, bilateral amygdala, left superior frontal gyrus, left ACC, bilateral parahippocampus, thalamus, left dorsal anterior insula, and the bilateral posterior insula.

Bao et al. (2018) found that patients with CD had lower resting-state FC between the hippocampus and the limbic system compared with the HCs. This research group also found that patients with CD revealed lower FC of the amygdala with the insula, parahippocampus, and the anterior middle cingulate cortex/dorsal ACC (Fan et al., 2019). A negative correlation between the disease duration and left amygdala-insula connectivity was observed (Fan et al., 2019). Two studies indicated abnormal FC between regions involved in the regulation of visceral sensation and pain processing. The FC, between the left superior frontal gyrus and the left precentral, middle temporal gyri, left ACC, left postcentral, middle frontal gyri, inferior frontal orbital cortex, and the right rolandic operculum, were found to be increased (Li L. et al., 2021), compared to the HCs. Qiu et al. (2022) found decreased FC between the left parahippocampus and bilateral thalamus, as well as the right parahippocampus and bilateral thalamus in CD patients. Taking left dorsal anterior insula and bilateral posterior insula as ROIs, Zhang et al. (2022) found insula-related FC differences were mainly located in the MCC, SMA, dorsolateral prefrontal cortex (dlPFC), caudate, ACC, S1, amygdala, and the parahippocampus/hippocampus, compared to the HCs. After controlling for anxiety and depression, Zhang et al. (2022) discovered there were no longer any FC differences between the left dorsal anterior insula and the ACC or between the right posterior insula and the dlPFC, ACC, and amygdala.

Resting-state functional connectivity networks are calculated from the degree of long-range second order temporal correlation pattern of activation signals in different brain regions (Perani, 2008). The presence of various sub-networks is revealed by subsequent study utilizing independent component analysis (Calhoun et al., 2009) or graph clustering approaches (Shi and Malik, 2000), particularly the dorsal attention, control/frontoparietal, salience, auditory, and default mode networks (Buckner et al., 2013). In this review, six studies were assessed using functional connectivity networks in patients with CD. Thomann et al. (2017) found that patients with CD presented abnormal connectivity in the DMN subsystems, which was associated with anxiety scores in CD patients. Hou et al. (2019) discovered increased FC in the central executive network (CEN) and DMN, which were found in the right precuneus and right posterior cingulate cortex, respectively, in the right middle frontal gyrus and right inferior parietal lobule. Another study found increased FC between the frontoparietal (FP) network and the salience network (SN), and decreased FC between regions within the DMN (Kornelsen et al., 2020). Li et al. (2022) explored the abnormal FC of intra- and inter-brain networks in CD patients, and found increased FC of the language network with the left middle temporal gyrus and decreased FC of the prime visual network with the left calcarine. Agostini et al. (2023) reported increased connectivity within the left FP network in inactive CD patients compared to active CD patients, decreased connectivity in the motor network in the active CD group compared to the HC group, and reduced connectivity in the motor network and in the language network in active CD patients compared to HCs. Thapaliya et al. (2023) observed increased connectivity in the FP network and visual network, as well as decreased connectivity in the SN and DMN. High abdominal pain levels were associated with lower connectivity in precuneus and parietal operculum and higher cerebellar connectivity. Greater disease duration was related with higher connectivity in middle temporal gyrus and planum (Thapaliya et al., 2023).

Detailed information was shown in Supplementary material.

## Discussion

In this study, we included 16 fMRI studies to analyze the difference in brain activity between patients with CD and healthy controls. Most (15 of 16) imaging studies included in this review were fully presented and contained all necessary entries. Through the descriptive analysis of this study, we found that the abnormal local brain activities, interested region related FC, and whole-brain functional connectivity networks in patients with CD at rest, mainly refer to brain regions in the frontal lobe, parietal lobe, insula, CC, amygdala, thalamus, PAG, hippocampal/parahippocampal cortex and SMA.

### The increased-decreased local brain activities in Crohn’s disease

In this study, the patients with CD showed abnormal fluctuations in extensive frontal and parietal brain regions, insula, CC, amygdala, thalamus, PAG, hippocampal/parahippocampal cortex, and SMA. The amygdala, frontal regions, ACC, and hippocampal/parahippocampal cortex are important nodes in brain emotional regulation circuit, and play an important role in emotional perception, experience, memory storage (Bermpohl et al., 2006; Yang et al., 2019). It has been reported that patients with irritable bowel syndrome (IBS) have abnormalities in the frontal regions, amygdala, frontal regions, ACC, and hippocampal/parahippocampal cortex (Hong et al., 2013; Ma et al., 2015). Animal research demonstrated that induced colitis increases circulating pro-inflammatory cytokines, which influence various brain areas, including the hippocampus, and generate anxiety- and depressive-like behaviors (Heydarpour et al., 2016; Haj-Mirzaian et al., 2017). In addition, correlation analysis supported an association between frontal regions, ACC and symptoms of anxiety and depression in patients with CD (Huang et al., 2022; Kong et al., 2022). Hence, chronic intestinal inflammatory stimulation in CD patients may result in aberrant resting state function in the core brain area of the emotional network.

The thalamus serves as the doorway to the cerebral cortex and is important in pain ascending transmission and pain communication (Basbaum et al., 2009). The PAG is a key neuronal substrate of the descending pain modulatory systems, involved in the processing and moderating of responses to somatic and visceral unpleasant stimuli (Staud, 2012). Several studies on chronic pain condition, such as fibromyalgia (Burgmer et al., 2009), chronic low back pain (Meier et al., 2017) and functional dyspepsia (Lee et al., 2016), have found abnormalities in one or more brain regions mentioned above. Additionally, it has been found that patients with ulcerative colitis (UC) have abnormalities in the thalamus (Agostini et al., 2011). Abnormally prolonged sensitization of visceral afferents in the gastrointestinal tract following acute inflammation can contribute to chronic pain (Gampierakis et al., 2019), this process is maintained by dysregulation of descending control emanating from the brain, where functional abnormalities in the thalamus and PAG have been observed (Agostini et al., 2011; Wei et al., 2015). In accordance with these studies, Bao C. et al. (2016) found hypoactivity at resting-state in thalamus and PAG, compared to HCs. The whole-brain analysis discovered an association between the ReHo values of the PAG and the daily pain levels in another investigation (Bao C. H. et al., 2016). In patients with CD, long-term chronic inflammation and visceral pain stimuli may transmit to cortical and subcortical areas and may cause aberrant resting state function in the main brain area of visceral sensation and pain processing.

### The altered FC and brain networks in Crohn’s disease

The cerebral cortex processes information by interacting various dispersed regions. Seven major brain networks have been identified utilizing connect omics to track functional connections, including the sensorimotor system, central executive network (CEN), default mode network (DMN), salience network (SN), dorsal attention network (DAN), visual system, and limbic/paralimbic system (Thomas Yeo et al., 2011). Most of the studies included in this review suggested the altered FC and brain networks mainly located in the DMN, CEN, and limbic regions. The DMN, is vital for maintaining resting brain function, which essentially include medial PFC, ACC/PCC, the precuneus, bilateral inferior parietal regions and other brain regions (Smallwood et al., 2021), and is thought to be involved in affective and cognitive self-referential processing (Smallwood et al., 2021). Thomann et al. (2017) found that increased FC within the ACC, left superior medial frontal gyrus and the MCC in CD patients compared to healthy controls. Hou et al. (2019) identified increased FC in the DMN subregions between the right precuneus and right PCC in CD patients compared to healthy controls. Thapaliya et al. (2023) showed decreased FC in the DMN in the parahippocampal gyrus. The three investigations discovered functional abnormalities in patients with CD that impacted the DMN and partially implied self-referential neural network dysregulation.

Unlike the DMN, the CEN exhibits activation in cognitive and emotional challenge activities. The CEN includes many regions, including the lateral prefrontal cortex, anterior cingulate cortex and inferior parietal lobule, etc., which are considered to support cognitive control, decision-making processes, and regulation of emotion (Seeley et al., 2007; Vincent et al., 2008). Several studies found alterations in the frontoparietal control system, including superior frontal gyrus, middle frontal gyrus, inferior frontal orbital cortex, inferior parietal, fusiform gyrus, PCC, postcentral gyrus, et al. (Hou et al., 2019; Li L. et al., 2021; Agostini et al., 2023; Thapaliya et al., 2023) in patients with CD. Abnormal FC between the frontal and inferior parietal lobule may reflect the disturbed functions of top-down control (Ciaramelli et al., 2008). On the other hand, excessive concerns about the gastrointestinal symptoms in patients with CD may further exacerbate visceral hypersensitivity and induce abnormalities in CEN. Additionally, patients with CD often experience psychological stress. Prolonged exposure to psychological stress in the context of CD may lead to modifications to brain regions responsible for the processing of emotions.

The limbic system refers to structures that are located at the border or edge of the hemispheres, plays an important role in emotional responses, as well as in learning, memory, and behavior (Pessoa and Hof, 2015; Rolls, 2019). The limbic structures include the cingulate cortex, the hippocampus, and the amygdala. The hippocampus is a key structure in neuroimmunological regulation, affecting humoral immunity and cellular immunity through the hypothalamic–pituitary–adrenal (HPA) axis and neurohumoral pathways (Lathe, 2001). For animal models in CD studies, hippocampal microglia activation, alterations in cytokine expression, and neurogenesis may lead to CNS excitability and behavioral changes (Riazi et al., 2015; Zonis et al., 2015; Heydarpour et al., 2016). The amygdala belongs to the limbic system and plays an important role in the regulation of emotion, visceral sensory processing, and pain processing (especially pain modulation and the emotional affective dimension of pain) (Berman et al., 2012; Neugebauer, 2015; Yang et al., 2019). The cingulate cortex is regarded as a special brain area that extends the emotional and memory domains and the internal neural network of the gastrointestinal tract (Vogt, 2013; Rolls, 2019; Kong et al., 2021; Yeung, 2021). The ACC receives inputs from the amygdala while the PCC has connections to the hippocampal (Rolls, 2019). Together, studies included found aberrant FC of the cingulate cortex, the hippocampus, and the amygdala with other functional brain networks in patients with CD, which may partially reflect the abnormal functions in visceral sensation, pain processing, and emotion regulation associated with CD.

### The altered brain activities and FC in active Crohn’s disease

Furthermore, four of the studies we included conducted analysis by stratifying patients according to their inflammatory activity. Compared with the inactive CD group, the active CD group exhibited significantly higher signals in the left ACC, the left superior frontal gyrus (Kong et al., 2022), the temporal superior regions, and the right temporal pole superior regions, and exhibited lower signals in the left occipital middle (Huang et al., 2022). In FC analysis, decreased connectivity was found in the superior parietal lobule in the active CD group (Agostini et al., 2023). The results of these studies confirmed a difference in resting state brain activity and FC between active CD patients and inactive CD patients. These altered brain regions may play crucial roles in attention, execution, memory, and emotional processing, which are associated with inflammatory factors (Tegeler et al., 2016; Shi et al., 2022). This suggests intricate connections between brain activity, disease activity, cognition, and emotion that require additional investigation in patients with CD.

Compared with the HC group, the active CD group demonstrated higher signals in the frontal superior medial region, frontal middle region, and lower signals in the supplementary motor area, the role of the temporal middle gyrus, and the postcentral gyrus (Huang et al., 2022). In FC analysis, decreased connectivity was found in motor-related areas (Agostini et al., 2023), in the SN and DMN (Thapaliya et al., 2023), and increased connectivity in the FP network and visual network (Thapaliya et al., 2023). This could be attributed to the significant contribution of chronic disease signal stimulation, in addition to the impact of active inflammation. The above-mentioned brain regions not only play a role in cognition, emotion, and pain processing, but also in somatosensory processing and voluntary motor control. The noxious signals of intestinal inflammation are transmitted to the brain via the brain-gut axis, resulting in changes in brain function. Similarly, the brain also controls the gastrointestinal tract through this channel. The abnormalities of these brain functions may result in dysfunction of cognition and emotion, disruption of the homeostatic responses to noxious stimuli, and visceral hypersensitivity.

### Psychological factors in Crohn’s disease

Psychological factors, including anxiety and depression, have been estimated to be more than 30% in patients with Crohn’s disease and ulcerative colitis (Neuendorf et al., 2016), which often lead to aggravation of gastrointestinal symptoms (Li J. et al., 2021). Thomann et al. (2017) showed a significant positive correlation between MCC and anxiety levels in patients with CD, not in HC. Zhang et al. (2022) reported that significant insula-related FC differences disappeared in CD patients when anxiety and depression were considered covariates. Huang et al. (2022) found abnormal resting-state brain activity was associated with psychological assessment scores in patients with active CD. These abnormal functional brain regions associated with psychological comorbidity were involved in the processing of negative emotions, which may reflect high sensitivity to negative emotions and disturbed visceral sensory processing in CD patients.

### Limitations

There are several limitations that need to be mentioned in this study. We did not conduct a meta-analysis due to significant differences in the analysis methods, inclusion, and exclusion criteria of the included studies. Findings of the studies included are heterogenous. Possible reasons for this include the following: First, the fMRI signal lacks the ability to effectively distinguish between function-specific processing and neuromodulation, as well as between bottom-up and top-down signals, which can potentially lead to confusion between excitation and inhibition; second, there are numerous methods available for analyzing fMRI data, each with its own advantages and disadvantages; third, the sample size in neuroimaging research is typically small, and there are insufficiencies in statistical efficiency; fourth, the original studies mainly recruited participants of Asian descent, resulting in a limited representation of subjects from around the world. The confounding effects resulting from genetic and cultural variations could not be assessed.

Furthermore, there were 4 studies that included active CD patients and inactive CD patients, and 2 studies did not mention the categories of included patients. The inconsistent CD categories may cause some unexpected confounds. Additionally, one study included assessed the brain changes before and after electro-acupuncture and moxibustion treatments, so much so that it was impossible to investigate the treatment effects systematically.

## Conclusion

Functional neuroimaging helps provide a better understanding of the underlying neuropathological processes in patients with CD. In this review, we summarize as follows: First, these findings indicate alterations in brain function in patients with CD, specifically affecting brain regions associated with pain, emotion, cognition, and visceral sensation; second, disease activity may have an impact on brain functions in patients with CD; and third, psychological factors may be associated with altered brain functions in patients with CD.

## Data availability statement

The original contributions presented in the study are included in the article/Supplementary material, further inquiries can be directed to the corresponding author.

## Author contributions

LY: Writing – original draft, Writing – review & editing. PH: Methodology, Writing – review & editing. LZ: Methodology, Writing – review & editing. KL: Writing – original draft, Writing – review & editing.
