# Supplementary material for: Altered resting-state brain functional activities and networks in Crohn’s disease: a systematic review
Source: Front Neurosci. 2024 Jan 24;18:1319359. doi: 10.3389/fnins.2024.1319359 (PMC10851432; doi:10.3389/fnins.2024.1319359)
Supplement: Supplementary file 1 [file Table_1.DOCX]

Table S1 Results summary of included studies

| Author  year | Statistical threshold | Main results of group comparisons | Symptom (measurement) | Correlation Analysis |
| --- | --- | --- | --- | --- |
| Bao et al. 2016a | *P* <0.05, corrected by FDR | ReHo: HC > CD with pain and without pain, insula, SMA, hippocampal/parahippocampal cortex and MCC;  HC < CD with pain and without pain, dmPFC | Pain intensity (VAS) | The ReHo values in the insula and MCC of patients with CD and with abdominal pain were  significantly negatively correlated with their daily pain scores;  For the whole-brain analysis, the daily pain scores negatively correlated with the ReHo values of the left insula, MCC, dlPFC, bilateral thalamus, and PAG and positively correlated with the right OFC. |
| Bao et al. 2016b | *P* <0.05, corrected by FDR | 1.ReHo:  CD>HCs, bilateral ACC, superior frontal medial cortex, middle frontal cortex, superior temporal pole, precuneus, right superior frontal cortex, inferior temporal cortex, angular gyrus, left middle temporal cortex, superior parietal cortex and middle occipital cortex；  CD < HCs, bilateral thalamus, insula, MCC, PCC, lingual gyrus, cerebellum, PAG, brainstem, left HIPP, SMA, postcentral gyrus, inferior frontal operculum cortex, right amygdala and superior temporal cortex；  2.Electro-acupuncture group：Increased ReHo values in the bilateral MCC, thalamus, the HIPP, the inferior frontal  cortex, the precentral cortex, the paracentral lobule and the superior temporal cortex, the left paraHIPP, the pallidum  and cerebellum, the right insula, the putamen, the postcentral cortex, the SMA, the lingual cortex and the  brainstem; Decreased ReHo values in the bilateral ACC, OFC, the inferior temporal cortex, the temporal pole and the middle occipital cortex, the left superior parietal cortex, the angular cortex and the cuneus, the right middle frontal cortex and the caudate nucleus；  3.Moxibustion group：Increased ReHo values in the bilateral MCC, pallidum, putamen, inferior frontal cortex, SMA, middle occipital cortex, cerebellum and brainstem, left PCC, paraHIPP/HIPP, paracentral lobule, angular cortex and fusiform cortex, right insula, precentral cortex and postcentral cortex；Decreased ReHo values in the bilateral middle temporal cortex and lingual cortex, left superior medial frontal cortex, inferior temporal cortex and temporal pole, right OFC, middle frontal cortex and the caudate nucleus. | Symptoms’ severity (CDAI score) | Electro-acupuncture group：The decrease in the CDAI score was significantly positively correlated with the decreases in the ReHo value in the ACC, the dlPFC and the temporal pole, and was negatively correlated with the increases in the ReHo value in the MCC, the thalamus, insula, the HIPP, the precentral and the postcentral cortex and SMA after treatment.  Moxibustion group：The decrease in the CDAI score was significantly positively correlated with the decreases in the ReHo value in the dlPFC, the dmPFC and the temporal pole, and was negatively correlated with the increases in the ReHo value in the MCC, the PCC, the precentral and postcentral  cortex and the SMA after treatment. |
| Thomann et al. 2017 | *P* < 0.05, corrected by FWE | CD > HCs, the anterior cingulate and left superior medial frontal gyrus (aDMN) and the middle cingulate cortex (pDMN) | Anxiety | MCC activity showed a significant association with anxiety scores in patients. |
| Bao et al. 2018 | *P* < 0.05, corrected by FDR | 1.ALFF: CD > HCs, HIPP/paraHIPP, anterior cingulate cortex, insula, superior frontal cortex and precuneus;  CD < HCs, S2, precentral gyrus, and medial prefrontal cortex.  2.FC: CD < HCs, between left HIPP and left inferior temporal cortex, and right middle cingulate cortex, HIPP, and fusiform area; between right HIPP and right inferior orbitofrontal cortex and left HIPP. |  |  |
| Liu et al. 2018 | *P* <0.05, corrected by FDR | CD < HCs, nodal graph metrics in the subcortical, sensorimotor, cognitive control and default-mode networks | Disease duration | The connectivity strength of putamen negatively correlated with CD duration in patients. |
| Hou et al. 2019 | *P* <0.05, corrected by FDR | 1.ECN-FC: CD > HCs, between the right middle frontal gyrus and right inferior parietal lobule.  2.DMN-FC: CD > HCs, between the right precuneus and right posterior cingulate cortex. | Verbal fluency and anxiety | The correlations between ECN/DMN and behavioral scores in each group were not significant. |
| Fan et al. 2019 | *P*< 0.05, corrected by FWE | CD patients exhibited decreased FC between the amygdala and insula, parahippocampus, anterior middle cingulate cortex/dorsal anterior cingulate cortex. | Disease duration | CD patients had negative correlation between the disease duration and amygdala-insula connectivity. |
| Kornelsen et al. 2020 | *P* <0.05, corrected by FDR | FC: CD > HCs, FC between the frontoparietal network and salience network, and decreased FC between nodes of the default mode network. |  |  |
| Li et al. 2021 | *P*< 0.05, corrected by FWE | 1.ALFF: CD > HCs, left superior frontal gyrus, anterior cingulate cortex, and supplementary motor area; CD < HCs, the left hippocampus.  2. ReHo: CD > HCs, the left anterior cingulate cortex, supplementary motor area, putamen, and the bilateral superior frontal gyri.  3.FC: CD > HCs, between the left superior frontal gyrus and the left precentral and middle temporal gyri; between the left anterior cingulate cortex and the left postcentral, middle frontal gyri, inferior frontal orbital cortex, and right rolandic operculum | Disease duration | There were no significant correlations. |
| Kong et al. 2022 | *P*< 0.05, Bonferroni-corrected | Patients in the active phase exhibited higher ALFF in the left ACC and the left superior frontal gyrus, medial superior frontal gyrus | depression | A positive relation exists between mWavelet-ALFF values of the left ACC and HADS-D scores |
| Li et al. 2022 | *P* <0.05, corrected by FDR | Compared with healthy controls, significantly increased connectivity was found between the language network and dorsal default mode network. | Disease duration | The FC values of the left calcarine within the prime visual network were negatively correlated with CD duration. |
| Qiu et al. 2022 | *P* <0.05, corrected by FDR | CD patients exhibited decreased FC between the left parahippocampus and bilateral thalamus, as well as the right parahippocampus and bilateral thalamus. | CDAI; disease duration; CRP; ESR and Calprotein | There were no significant correlations between FC and clinical characteristics. |
| Huang et al. 2022 | *P* <0.05, corrected by GRF | Compared with HCs, the active CD patients exhibited higher ReHo values in the frontal superior medial, frontal middle and lower values in the postcentral, supplementary motor area, and temporal middle. Inactive CD patients exhibited higher ReHo values in the frontal middle and lower ReHo values in the precentral, postcentral and putamen. | Psychological assessment; ESR; obsessive-compulsive,  depression, bigoted scores, systemic symptoms | Several brain regions were significantly correlated with clinical characteristics. |
| Zhang et al. 2022 | *P* <0.05, corrected by FDR | Taking the left dorsal anterior insula and bilateral posterior insula as regions of interest, differences were observed in the FC of the ROI with several regions between the two groups. | CDAI | CD patients exhibited significant negative correlations between the CDAI and FC of the bilateral paraHIPP/HIPP and the left dAI and right PI |
| Agostini et al. 2023 | *P*< 0.05, corrected by FWE | 1. Inactive CD > active CD, the superior parietal lobule  2. active CD < HCs, the motor-related areas  3. Inactive CD < HCs, the cingulate gyrus and the precuneus and the cingulate gyrus |  |  |
| Thapaliya et al. 2023 | *P< 0.05, corrected by TFCE* | 1. active CD > HCs, the inferior temporal gyrus and lateral occipital cortex (Visual Network), the OFC (medial Visual Network), and the inferior temporal gyrus, occipital fusiform gyrus, thalamus, caudate, PCC, postcentral gyrus and lingual gyrus (FPN)  2. active CD < HCs, the PHG and cerebellum (DMN), the cerebellum, lingual gyrus and postcentral gyrus (SN), and the occipital fusiform gyrus and cerebellum (Cerebellar Network) | Disease duration, abdominal pain score | Greater abdominal pain scores were associated with lower  connectivity in the precuneus (visual network) and parietal operculum (salience network), and higher connectivity in the cerebellum (frontal network). Greater disease duration was associated with greater connectivity in the middle temporal gyrus and planum temporal (visual network). |

fMRI, functional magnetic resonance; BOLD, blood oxygenation level-dependent; ReHo, regional homogeneity; FC, functional connectivity; FNC, functional network connectivity; ALFF, amplitude of low frequency fluctuation; ROI, Region of interest; CD, Crohn's disease; HCs, health controls; FDR false discovery rate, FWE familywise error rate; MCC, midcingulate cortex; ACC, anterior cingulate cortex; SMA, supplementary motor area; dmPFC, Dorsomedial prefrontal lobe; dlPFC, dorsolateral prefrontal cortex; HIPP, hippocampus; PCC, posterior cingulate cortex; dAI, dorsal anterior insula; PI, posterior insula; OFC, orbitofrontal cortex; DMN, default mode network; ECN, executive control network; VAS, visual analogue scale; HADS-D, hospital anxiety and depression scale, depression score; CDAI, Crohn’s Disease Activity Index; CRP, C-reactive protein; ESR, erythrocyte sedimentation rate

Search Strategy:

PubMed

| Search | Query | Results |
| --- | --- | --- |
| #1 | CD[Title/Abstract] OR Crohn’s disease [MeSH Terms] | 203,629 |
| #2 | (Magnetic Resonance Imaging [MeSH Terms]) OR (MRI[Title/Abstract]) OR (fMRI [Title/Abstract]) OR (Functional MRI[Title/Abstract]) OR (rs-fMRI [Title/Abstract]) OR (resting-state fMRI [Title/Abstract]) | 675,767 |
| #3 | (BOLD[Title/Abstract]) OR (Blood oxygenation level dependent [Title/Abstract]) | 14,775 |
| #4 | #1 AND (#2 OR #3) | 3,069 |

Web of science

1. TS= (“Crohn’s disease”)

2. TS= (Magnetic Resonance Imaging) OR (MRI) OR (fMRI) OR (Functional MRI) OR (rs-fMRI) OR (resting-state fMRI) OR (BOLD) OR (Blood oxygenation level dependent)

1 AND 2

Result: 1,726

EMBASE

Embase <1966 to 2023 December 15>

1# Magnetic Resonance Imaging ab,ti. 351,663

2# MRI ab,ti. 545,993

3# fMRI ab,ti. 72,362

4# Functional MRI ab,ti. 19,746

5# rs-fMRI ab,ti. 4,198

6# resting-state fMRI ab,ti. 8,354

7# BOLD ab,ti. 20,553

8# Blood oxygenation level dependent ab,ti.3,880

9# 1# OR 2# OR #3 OR #4 OR #6 OR #7 OR 8# 768,613

10# Crohn’s disease ab,ti 6,054

11# 9# AND 10# 244
